# Antitumor and Angiostatic Activities of the Antimicrobial Peptide Dermaseptin B2

**DOI:** 10.1371/journal.pone.0044351

**Published:** 2012-09-20

**Authors:** Hanneke van Zoggel, Gilles Carpentier, Célia Dos Santos, Yamina Hamma-Kourbali, José Courty, Mohamed Amiche, Jean Delbé

**Affiliations:** Laboratoire de Recherche sur la Croissance Cellulaire, la Réparation et la Régénération Tissulaires, Université Paris Est – Créteil, Créteil, France; The University of New South Wales, Australia

## Abstract

Recently, we have found that the skin secretions of the Amazonian tree frog *Phyllomedusa bicolor* contains molecules with antitumor and angiostatic activities and identified one of them as the antimicrobial peptide dermaseptin (Drs) B2. In the present study we further explored the *in vitro* and *in vivo* antitumor activity of this molecule and investigated its mechanism of action. We showed that Drs B2 inhibits the proliferation and colony formation of various human tumor cell types, and the proliferation and capillary formation of endothelial cells *in vitro*. Furthermore, Drs B2 inhibited tumor growth of the human prostate adenocarcinoma cell line PC3 in a xenograft model *in vivo*. Research on the mechanism of action of Drs B2 on tumor PC3 cells demonstrated a rapid increasing amount of cytosolic lactate dehydrogenase, no activation of caspase-3, and no changes in mitochondrial membrane potential. Confocal microscopy analysis revealed that Drs B2 can interact with the tumor cell surface, aggregate and penetrate the cells. These data together indicate that Drs B2 does not act by apoptosis but possibly by necrosis. In conclusion, Drs B2 could be considered as an interesting and promising pharmacological and therapeutic leader molecule for the treatment of cancer.

## Introduction

Cancer is a disease that nowadays seems to become the most important cause of death. Nevertheless, in the last few decades significant progresses has been made in terms of understanding and treatment of this disease. Depending on the diagnosis, origin and state of the cancer several managing options exist, including surgery, radiation therapy, and chemotherapy. Unfortunately, concerning chemotherapy, the activity of most chemotherapeutic agents counteract also against the healthy fast-dividing cells of the body, such as blood cells and the cells lining the mouth, stomach, and intestines. Furthermore, they can depress the immune system and cancer cells frequently develop resistance to many anti-cancer drugs that greatly reduces their therapeutic usefulness [1], [2]. Other common side effects include fatigue, tendency to bleed easily, gastrointestinal distress, rapid weight loss, or occasionally in weight gain, and temporary hair loss.

Due to the constant need to improve or find new therapeutic agents against cancer and especially those that are able to evade drug resistance and other significant side-effects, peptides have become one of the new research targets. A group of interesting natural-source peptides consists of antimicrobial peptides (AMPs). They have been isolated from a wide variety of organisms as single-celled microorganisms, insects, plants, birds, fish, amphibians, and mammals, including humans [3], [4]. AMPs were initially discovered due to their role in the clearance of microorganisms [5]–[8] and are released in response to infection by a different regulatory process [5], [9], [10]. AMPs mainly target the plasma membrane and they quickly kill the microorganisms *in vitro* (within a few minutes). This killing activity covers a wide spectrum including Gram-positive and -negative bacteria, fungi and protozoa without being cytotoxic to mammal cells at the doses that have shown antimicrobial activity [11], [12]. Besides, AMPs kill pathogens that are known to be multi-resistant to conventional antibiotics and since it is very difficult for a microorganism to radically change the organization of its plasma membrane, potentially low levels of induced resistance occur compared to conventional antibiotics [13], [14].

Interestingly, in addition to the above named activities, a growing number of studies showed that some of the cationic AMPs exhibit a broad spectrum of cytotoxic activity against cancer cells. AMPs that are able to kill cancer cells can be placed into two categories [15]: the first contains AMPs that are highly potent against bacteria and cancer cells but not against normal mammalian cells, for example insect cecropins [16], [17] and magainins [18]–[20]. The second group is composed of AMPs that are cytotoxic for bacteria, cancer cells, and normal mammalian cells. Some examples of this last group include the bee venom melittin [21], tachyplesin-II isolated from horseshoe crab [22], human neutrophil defensins [23], and human LL-37 [24]. Nevertheless, many AMPs do not possess any anticancer activity [19], [25]–[27].

Recently, we have reported significant antitumor activity of the AMPs dermaseptin (Drs) B2 and Drs B3 against human cancer prostate cells PC3 *in vitro* [28]. Drs B2 and Drs B3 are members of the Drs B family and are derived from the skin secretions of the South American frog *Phyllomedusa bicolor*. They have high membrane-lytic antibacterial activity whereby they quickly kill Gram-positive and Gram-negative bacteria, yeast, protozoa and filamentous fungi, and have little or no haemolytic activity [29]–[32]. As Drs B2 is regarded as the most abundant member and the most active peptide of the B family with a minimal inhibitory concentration in micromolar range [31], [33]–[35] we further investigated the antitumor activity of this peptide. Drs B2, also known as adenoregulin, [36] is an α-helical amphipathic polycationic polypeptide (NH_2_-GLWSKIKEVGKEAAKAAAKAAGKAALGAVSEAV-CONH_2_) with a molecular mass of 3180 Da.

The aim of this study was to analyze the antitumor efficacy of Drs B2 on a wide range of human tumor cells *in vitro* and to evaluate its antitumor activity in a PC3 tumor xenograft mice model. In order to explore the possible mechanism of action of Drs B2 on tumor PC3 cells, experiments related to cell viability, cell death, membrane and/or mitochondrial integrity are performed. Additionally, immunostaining experiments using an anti-Drs B2 polyclonal antibody are accomplished to localize where Drs B2 is acting on and eventually inside the cells.

## Results

### Effect of Drs B2 on proliferation of tumor and non tumor cells

Several concentrations of Drs B2 were tested on the proliferation of various tumor and non tumor cells from human origin. The dose-dependent activity of Drs B2 on cell viability curves is resumed in Table 1. Results are expressed in growth inhibition 50% (GI50) which indicates the peptide concentration that inhibit 50% of the cell growth. The data reveal that Drs B2 is most active against the proliferation of tumor adherent and non-adherent cell lines and shows GI50 values in the low micromolar range. The highest observed GI50 value of 8 µM is related to the inhibition of breast carcinoma MDA-MB231 cells after treatment with Drs B2. Also the proliferation of the non tumor but immortalized cells LB-EBV, HTK and PNT1A was inhibited by this peptide. At a maximum tested concentration of 15 µM, Drs B2 did not affect proliferation of the tested human primary normal cells G1947 and FD.

**Table 1:** Effect of Drs B2 on proliferation of human tumor and non tumor cells.

	Cell name	Cell type	GI50
			[µM of Drs B2 ± SD]
tumor	PC3	prostate adenocarcinoma	1.24±0.23
	LNCAP	prostate adenocarcinoma (adrenogen sensitive)	0.31±0.15
	DU145	prostate carcinoma (non adrenogen sensitive)	0.91±0.04
	MDA-MB231	mammary carcinoma	8.06±0.50
	RAJI	B-lymphoma	2.57±0.75
immortalized	LB-EBV	B-lymphoblastoids, transformed by Epstein-Barr virus	1.09±0.56
	HTK	corneal fibroblasts	1.80±0.40
	PNT1A	prostatic cell line (non adrenogen sensitive)	0.38±0.05
primary	G1947	stromal prostate fibroblasts	*
	FD	skin fibroblasts	*

All the cells used are from human origin, the names of cell lines or types and their tissues of origin are indicated. Proliferation of each cell type was performed in plastic 24 wells plates (1.91 cm^2^; cell density of 1×10^4^ cells/well/0.5 mL). On the first, third and fifth day after plating the cells were treated with Drs B2 at different concentrations ranging from 0 till 15 µM. Twenty four hours after the last treatment cell counting was performed with crystal violet staining for adherent cells and with a Malassez Hemacytometer for non-adherent cells. Results are represented in peptide concentration inhibiting 50% of the cell growth GI50 ± SD of at least three determinations.

* No cytotoxicity observed when cells were treated with a maximal concentration of 15 µM of Drs B2.

### Effects of Drs B2 on PC3 and MDA-MB231 colony formation *in vitro*

**Figure 1:**
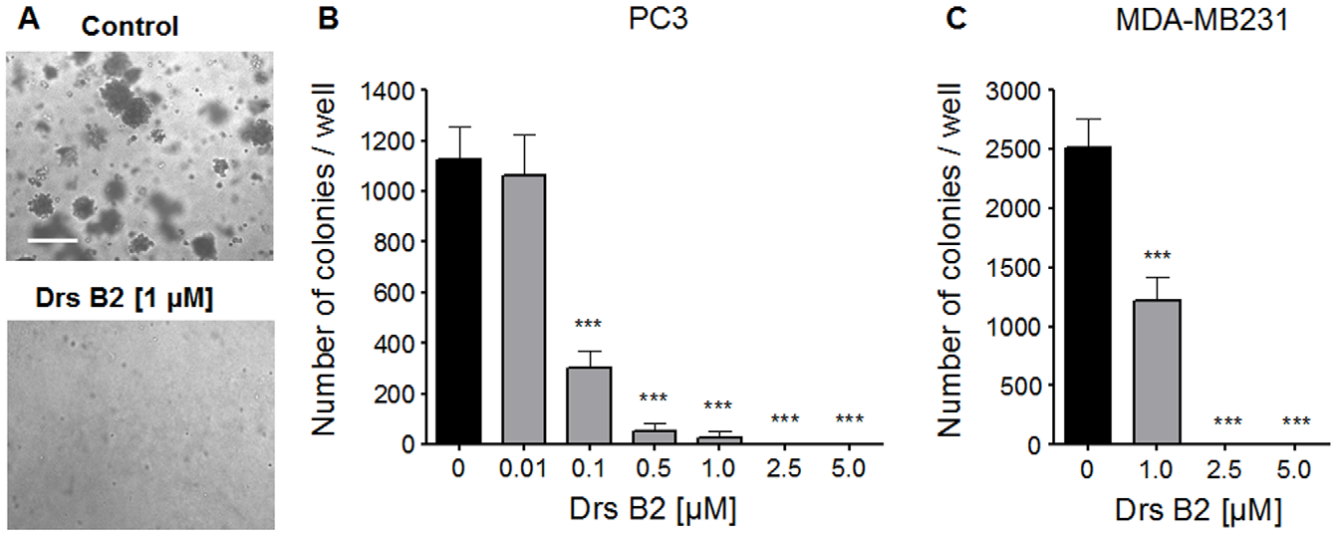
Effect of Drs B2 on colony formation of the tumor cell lines PC3 and MDA-MB231. The effect of Drs B2 on colony formation of the tumor cell lines PC3 and MDA-MB231 was tested into soft agar (12 wells plates, 3.8 cm^2^; cell density of 1×10^4^ cells/well/1 mL). Directly after plating and every 48 hours during 10 days cells were treated with different concentrations of Drs B2. After 10 days the colonies per field (1 mm^2^), bigger than 100 µm were counted. ***A*** *)* Photos of PC3 colony formation; 10× magnification; bar = 200 µm. ***B*** *)* PC3 colony quantification and ***C*** *)* MDA-MB231 colony quantification are expressed in the number of colonies per well. Results represent the mean of five fields of 1 mm^2^ per well ± SEM of three determinations. *** p<0.001 versus control (0 µM).

Since Drs B2 showed a lower GI50 on tumor cells than on non tumor cells, Drs B2 was further studied to explore its activity against cancer cells and on colony formation of PC3 and MDA-MB231 cells *in vitro*. As shown in Figures 1A and 1B, treatment with Drs B2 of 0.1 µM significantly inhibited PC3 cell colony formation with more than 70% compared to untreated conditions. Concentrations above 2.5 µM showed a complete inhibition of colony formation. Similarly, MDA-MB231 colony formation was inhibited by Drs B2 (Figure 1C). Treatment with 1 µM inhibited colony formation of about 50% compared to untreated cells. Colony formation was completely inhibited when treated with 2.5 µM or higher. These experiments confirmed the anti-proliferative activity of Drs B2 on the PC3 and MDA-MB231 human tumor cell lines.

### Effects of Drs B2 on endothelial cell proliferation and differentiation *in vitro*

**Figure 2:**
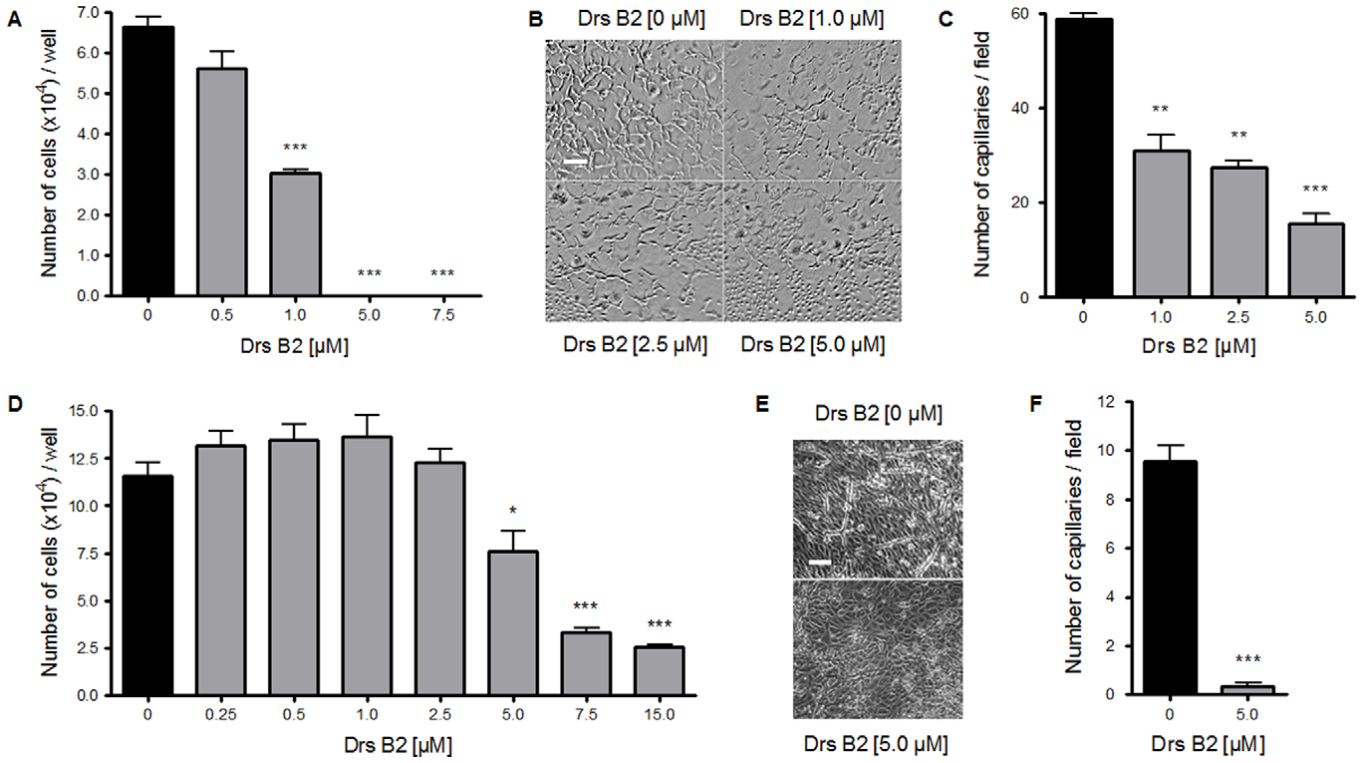
Effect of Drs B2 on the proliferation and differentiation of endothelial cells. ***Effects of Drs B2 on human umbilical vein endothelial cell (HUVEC) A) proliferation and B, C) differentiation. B)*** Differentiation of HUVEC cells was performed into 24 wells plates (1.91 cm^2^; cell density of 4×10^4^ cells/well/0.5 mL) pre-coated with Matrigel™ (1/3 diluted). Cell culture medium and Matrigel™ were supplemented with FGF-2 (20 ng/mL). Directly after plating cells were treated with Drs B2 (1, 2.5 and 5 µM). Twenty four hours after Drs B2 treatment tube formation was observed with a phase contrast microscope, photographed (expansion 50×; bar: 250 µm) and counted. ***C*** *)* Quantification of HUVEC differentiation is expressed in the number of pseudo capillaries per field. Results represent the mean of two fields ± SEM of three determinations. ***Effects of Drs B2 on adult bovine aortic endothelial (ABAE) cell D) proliferation and E, F) differentiation. E)*** Differentiation of ABAE cells on collagen induced by FGF-2 (20 ng/mL) was tested with the Montesano test (12 wells plates, 3.8 cm^2^; cell density of 2×10^5^ cells/well/1 mL). Directly and forty eight hours after plating cells were treated with Drs B2 (5 µM). Forty eight hours after the last treatment pseudo capillaries were observed with a phase contrast microscope and counted. Capillary formation is observed with a phase contrast microscope, photographed (expansion 10×; bar 100 µm) and counted. ***F*** *)* Quantification of ABAE differentiation is expressed in the number of pseudo capillaries per field. Proliferations assays (**A, D**) were performed as described in Materials and Methods. Results are represented in the number of cells per well and represent the mean ± SEM of at least 3 determinations. For differentiation assays, results represent the mean of five fields ± SEM of three determinations. * p<0.05; ** p<0.01; *** p<0.001 versus control (0 µM).

Since we previously reported that partially purified peptides fractions from the skin secretions of the frog *Phyllomedusa bicolor* inhibited the proliferation and differentiation of ABAE cells [28], the effect of Drs B2 on human endothelial cell was also explored. As shown respectively in Figures 2A and D, the proliferation of the HUVEC and ABAE cells was inhibited by Drs B2 in a dose dependant manner. The angiostatic abilities of Drs B2 were observed by testing its effect on two *in vitro* angiogenesis models using ABAE cells on collagen according Montesano and using HUVEC on Matrigel™. In control conditions in the presence of FGF-2, HUVEC cells have formed a complete linked capillary network onto Matrigel™ 24 hours after plating (Figures 2B and C). Compared to the untreated control conditions, treatment with Drs B2 inhibited HUVEC pseudo capillary formation at 24 hours after treatment in a dose-dependent manner from 1 to 5 µM. These results are confirmed by the anti-angiogenic activity of Drs B2 on ABAE differentiation on collagen according to the Montesano model (Figures 2E and F). In the presence of FGF-2 ABAE cells formed capillary tubes onto collagen 4 days after plating whereas treatment with 5 µM Drs B2 completely inhibited the formation of these tubes (Figure 2F). Observations of both endothelial cell differentiation models suggest that Drs B2 disturbs the formation of capillaries without having toxic activity towards undifferentiated cells attached onto the layers.

### Effects of Drs B2 on tumor growth *in vivo*

**Figure 3:**
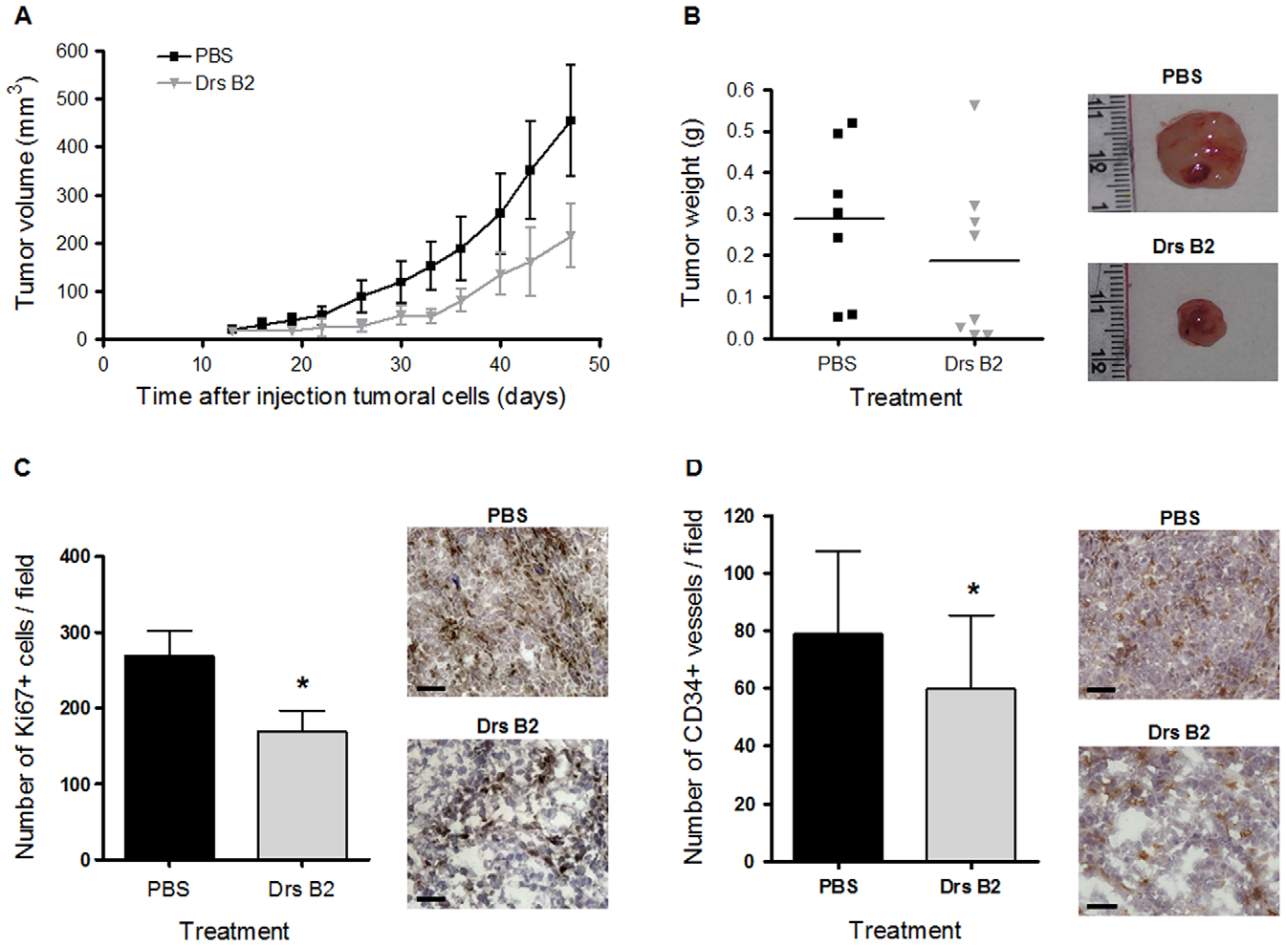
Effect of Drs B2 on tumor growth *in vivo*. Treatment of xenografted nude mice was started thirteen days after s.c. PC3 cell injection. Experimental group (n = 8) was treated six times per week p.t. with 100 µL Drs B2 (2.5 mg/kg body weight). Control group (n = 7) was treated six times per week p.t. with 100 µL of PBS. ***A*** *)* The effect of Drs B2 on tumor size versus time of treatment. After 47 days of treatment the mice were sacrificed, body weight was observed; tumors were isolated, ***B*** *)* weighted and stored at −80°C. ***C*** *)* PC3 tumor proliferation was evaluated by Ki67 staining of frozen tissue sections. Proliferation was quantified by image J software analysis of Ki67 positive stained cells on the whole tumor section. The data are mean areas ± SD obtained from 6 control mice and 4 mice treated with Drs B2. Scale bar, 50 µm. *p<0.05 versus control (PBS). ***D*** *)* Tumor vessel formation was observed with anti CD34 antibodies. Angiogenesis was quantified by image analysis of CD34 positive stained endothelial cells. The data are mean areas ± SD obtained from 6 control mice and 4 mice treated with Drs B2. Scale bar, 50 µm *p<0.05 versus control (PBS).

As shown in Figure 1, Drs B2 has the capacity to completely inhibit the colony formation of MDA-MB231 and PC3 cells *in vitro*. Since PC3 cells present an average sensibility to Drs B2 and are able to form tumor in mice, these prostate adenocarcinoma cells were chosen as the representative cell line for the remainder of this investigation. Consequently, the effect of Drs B2 was evaluated *in vivo* via a xenograft model in nude mice using this type of human tumor cells. Thirteen days after injection of PC3 cells one single tumor of around 25 mm^3^ was developed where after treatments were started. Figure 3A shows the tumor volume versus time of treatment. At the end of the experiment Drs B2 inhibited tumor growth with more than 50% compared to tumors treated with PBS. However, as a result of the large dispersion of the tumor volume in mice from each group, no significance was obtained when using the two-tailed t-test. In two mice, Drs B2 completely inhibited tumor growth. The inhibitory patron of tumor weight due to Drs B2 was equal to that of the tumor size (Figure 3B); but for similar reason, this inhibition was not significant when using the same statistical analysis. Moreover, compared to non-tumor-bearing mice, total blood cell count was not significantly changed when treated with Drs B2 (data not shown). Additionally, no mortality and no long-lasting side effects nor any signs of weakness, diarrhea, appetite or lethargy were observed during this experiment.

In order to further define the effect of Drs B2 on tumor proliferation *in vivo*, Ki67 labeling was performed on tumor sections from each group. As shown in Figure 3C, the proliferation levels were significantly lower (decreased with 36%) in Drs B2-treated mice as compared to the control group. The slight inhibition observed in the Drs B2-treated group could be related to the large dispersion of the tumor weight observed in Figure 3B. Finally, the effect of Drs B2 on tumor vascularization was investigated using CD34 labeling on tumor sections to quantify tumor blood vessels. Compared to untreated tumors, peri-tumoral (p.t.) injections of Drs B2 slightly, but significantly decreased (with 24%) the number of vessels (Figure 3D). These results suggested that the antitumor effect of Drs B2 could also act through a direct inhibition of the associated angiogenesis and confirm the angiostatic activity of Drs B2 observed *in vitro* as shown in Figure 2.

### Mechanism of Action of Dermaseptin B2

**Figure 4:**
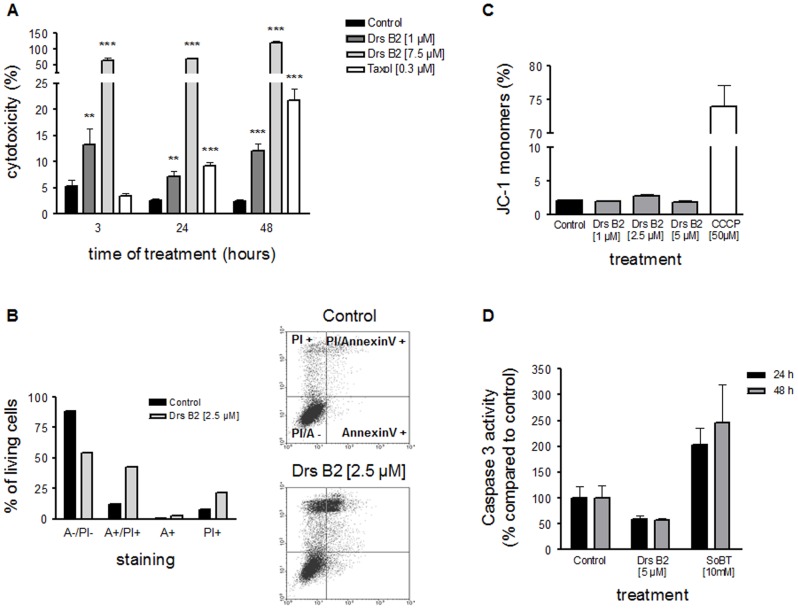
Mechanism of action of Drs B2 on tumor PC3 cells. ***A*** *)* ***Cytotoxic effect of Drs B2 on PC3:*** PC3 were cultured (FBS; 5%) in plastic 96 wells plates (0.035 cm^2^; cell density of 1.5×10^3^ cells/well/0.1 mL). Twenty four hours after plating cells were single treated with Drs B2 (1 or 7.5 µM). Taxol® (0.3 µM) is used as an internal positive control. LDH release was measured with CytoTox 96 kit at different time points after treatment. Results are expressed in percentage of cytotoxicity versus time of treatment in hours. Results represent the mean ± SEM of 3 determinations. * p<0.05; ** p<0.01 *** ; p<0.001 versus control (0 µM) of the related time point. ***B*** *)* ***PC3 cell viability after Drs B2 treatment:*** PC3 cells were treated with Drs B2 (2.5 µM). Twenty four hours after treatment cells where double stained with FITC-Annexin-V and PI and analyzed by flow cytometry. The cell viability of PC3 cells was observed by measuring the amount of Annexin-V and PI negative and positive cells. Counter diagrams show an example of FITC-Annexin-V/PI double staining after PC3 treatment with Drs B2 (2.5 µM) during 24 hours. Graph represents the mean amount of cells per staining in %. PI^−^/A^− = ^PI and Annexin-V negative; PI^+ = ^PI positive and Annexin-V negative; A^+ = ^Annexin-V positive and PI negative; PI^+^/A^+ = ^PI and Annexin-V positive. ***C*** *)* ***Effect of Drs B2 on mitochondrial membrane potential of PC3:*** PC3 cells were treated with Drs B2 (1, 2.5 and 5 µM) during 1 hour where after stained with JC-1 dye and analyzed by FACS. Change in mitochondrial membrane potential (ΔΨm) of PC3 cells was observed by measuring the amount of JC-1 monomers and JC-1 aggregates. Results are expressed in % of JC-1 monomers. Results represent the mean ± SEM of at least 2 determinations. ***D*** *)* ***Caspase-3 activity after Drs B2 treatment:*** PC3 cells were treated with Drs B2 (5 µM) and SoBT (10 mM) during 24 or 48 hours. Caspase-3 activity was measured on protein lysate by using specific fluorescent caspase-3 substrate. The results are expressed in % of activity found in untreated cells and represent the mean ± SEM of at least 2 determinations performed with independent cell cultures.

To obtain more information about how Drs B2 acts on PC3 tumor cells, the mechanism of action of this molecule was studied. Since the anti-proliferative effect of Drs B2 on tumor cells resembled more to a killing-like effect, experiments concerning the cytotoxic effect, the induction of apoptosis or necrosis and the effect of this peptide on mitochondria were investigated. To confirm this assumption, first, the cytotoxic effect of Drs B2 was tested. PC3 cells were treated with 1 and 7.5 µM of Drs B2 corresponding respectively to the GI50 and GI100 concentrations and the amount of released cytoplasmic LDH into the medium was measured at different time points (Figure 4A). Cells treated with Taxol® were used as an internal positive control. Half an hour after treatment of PC3 cells with 1 µM Drs B2 showed a significant cytotoxic effect (more than 5%) compared to the untreated control group (results not shown). Three hours after treatment with 1 and 7.5 µM of Drs B2, PC3 cells released respectively 15 and 80% of the total LDH into the medium. These percentages did not increase when PC3 cells were treated during longer times, 24 and 48 hours. These results contrasted with the longer time cytotoxic effect of Taxol® and indicated that Drs B2 is already cytotoxic for PC3 cells in a short period after treatment, within 3 hours.

The cytotoxic effect of Drs B2 was further investigated by observing cell viability and to see if apoptosis of cells could be implicated. PC3 cells were treated with Drs B2 for 24 hours, double labeled with PI and Annexin-V and analyzed by flow cytometry. Figure 4B shows the amount of cells per staining in % of the total cell number. In untreated conditions around 10% of the PC3 cells showed Annexin-V/PI positive staining. Treatment of these cells with 2.5 µM Drs B2 increased the amount of PI positive cells (>20%) and Annexin-V/PI positive cells (>40%) compared to the untreated control group (respectively 8% and 12%). Longer time of exposure to Drs B2, 72 hours, did not change these amounts. It has to be mentioned that no clear distinguish could be made between the PI positive and the Annexin-V/PI positive populations. Since no cells were observed that only present Annexin-V positive staining, it could be that the Annexin-V/PI positive mentioned population represents more the PI positive cells. To confirm these results specific apoptotic markers have to be used whereby a more clear distinguish can be made between the cells that undergo apoptosis or those that altered into the late apoptotic/necrotic state. It is noteworthy that the morphology of PC3 cells was changed after Drs B2 treatment. These cells were bigger and showed increased granularity compared to untreated conditions (data not shown).

To investigate if the cytotoxic effects of Drs B2 were related to changes in the mitochondrial transmembrane potential, the intensity and shift of fluorescence emission of the JC-1 dye were monitored by flow cytometry. PC3 cells were treated with different concentrations of Drs B2 during 1 hour where after stained with JC-1 and observed with flow cytometry (Figure 4C). CCCP, an uncoupler of oxidative phosphorylation [37] known to trigger mitochondrial membrane potential was used as a positive control. In a parallel experiment, cells were stained with PI and results indicate that most cells were viable at the time of JC-1 observation (results not shown). Treatment of PC3 cells with 1, 2.5 and 5 µM of Drs B2 during 1 hour showed no marked change in JC-1 monomers in the cytosol (green fluorescence) versus JC-1 aggregates in the mitochondria (red fluorescence) compared to untreated cells. In cells exposed to 50 µM of CCCP during 24 hours, a decline in red fluorescence was observed as a result of progressive uploading of JC-1 from the depolarized mitochondria, thus resulting in brighter green cell cytoplasm. Treatment with 1 and 2.5 µM of Drs B2 during 24 hours did not show such fluorescent shift (results not shown). Taken together, these results indicate that Drs B2 did not affect the mitochondrial membrane potential (ΔΨm) in PC3 cells and suggest that the mitochondria are not a target of Drs B2.

Early events of apoptosis could lead to the activation of the proteasome that plays an important and diverse role in the regulation of the apoptotic process and the activation of caspase proteases, including caspase-3. Therefore, caspase-3 activity was measured when PC3 cells were exposed to 5 µM of Drs B2 during 24 and 48 hours. Cells were lysed, proteins were isolated, quantified and an equal amount of proteins was used for each sample. As control, sodium butyrate (SoBT) was used. The results obtained 24 and 48 hours after the different treatments are shown in Figure 4D. Treatment with Drs B2 did not increase the activity of caspase-3, but surprisingly a slight, non significant decrease was observed. Cells exposed to SoBT showed more than double caspase-3 activity than observed in untreated conditions, indicating that the internal test conditions were well established.

**Figure 5:**
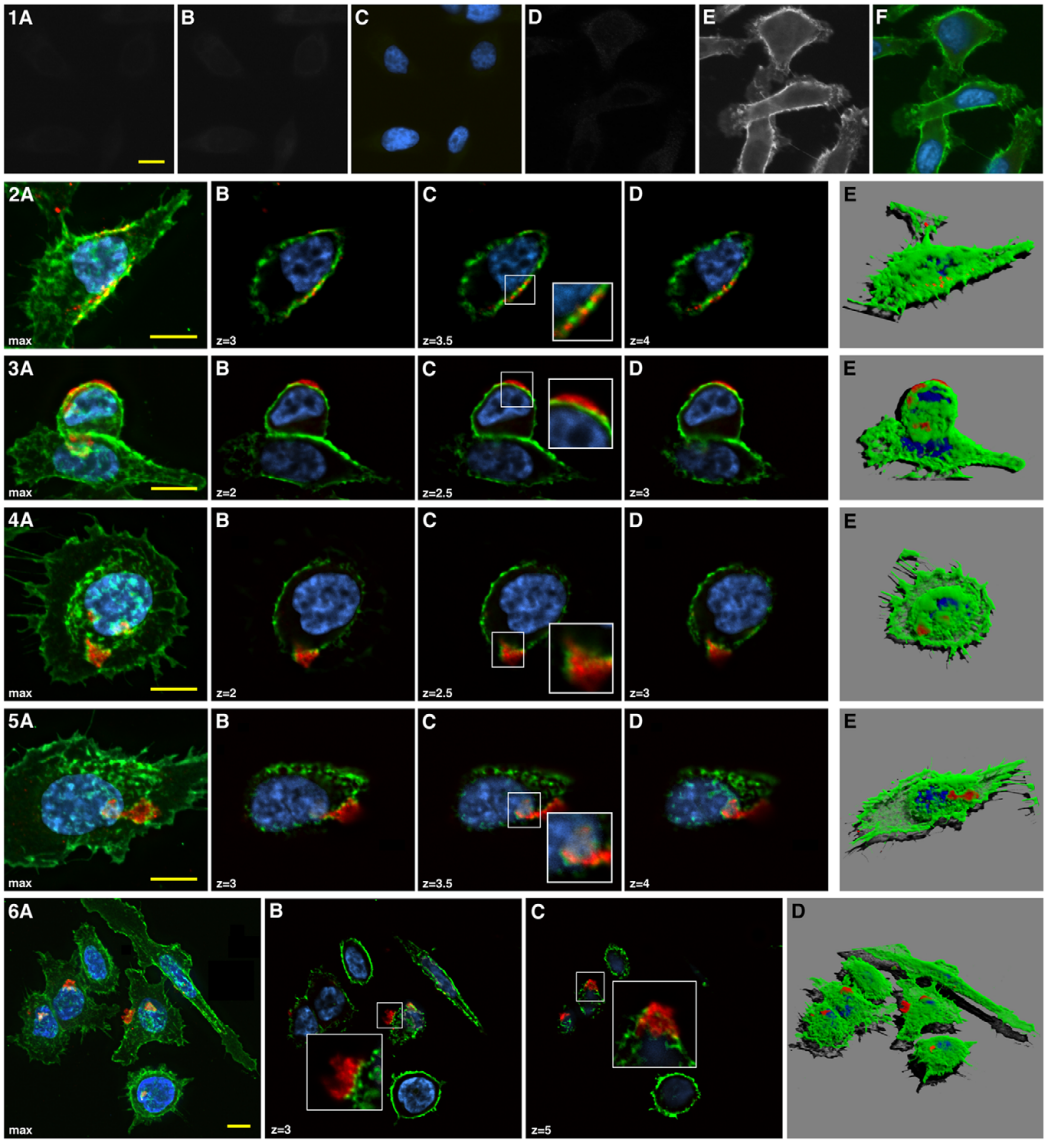
Immunostaining of PC3 cells after Drs B2 treatment. PC3 cells were treated with 2.5 µM of Drs B2 during 1 hour where after fixed and stained with anti-Drs B2 polyclonal purified rabbit IgG. Drs B2 is visualized by using Fluo 546-donkey rabbit antibody (red), membranes by CD147 labeled with FITC-AffiniPure Goat Mouse antibody (green) and nuclei by DAPI (blue). Confocal fluorescence images were acquired using an IX81 inverted Olympus microscope (60× oil-immersion NA 1.25 objective) equipped with a DSU spinning disk confocal system (Olympus; Rungis, France), coupled to an Orca R2 CCD camera (Hamamatsu Corporation; Japan). Images were arranged using the image processing software ImageJ, and the program Zoom in Images and Stack. Panels 1A–C present secondary antibody controls 1A) for the Drs B2 detection by fluo 546-donkey rabbit antibody and 1B) for the CD147 detection by FITC-AffiniPure Goat Mouse antibody. Panel 1C corresponds to the composite image of 1A and 1B plus the DAPI labeling of the same field. Panels 1D–F show labeling controls for 1D) the Drs B2 detection by anti Drs B2 and fluo 546-donkey rabbit antibodies in absence of Drs B2 treatment, and for 1E) the CD147 detection by anti CD147 and FITC-AffiniPure Goat Mouse antibodies. Panel 1F corresponds to the composite image of 1D and 1E plus the DAPI labeling of the same field. Panels 2A, 3A, 4A, 5A and 6A present the maximum projection image of the composite RGB stacks along the z axis. Panels 2B–D, 3B–D, 4B–D, 5B–D and 6 B–C show the confocal image series of the same region of interest wherein z represent the distance from the base of the cell in µm (insets zoom factor = 2). Panels 2E, 3E, 4E, 5E, 6D present the 3D volume rendering of the cells from the composite RGB stacks prepared by using the software FreeSFP. Scale bar: 10 µm.

Since the activity of Drs B2 seems to be an early event we analyzed whether Drs B2 acts on tumor PC3 cell membrane and eventually enters into these cells. For these experiments EMMPRIN/CD147, a transmembrane glycoprotein ubiquitously expressed by normal and tumor epithelial cells, was used as marker of the plasma membrane [38]. The results are depicted in Figure 5. During confocal microscopic examination no significant signal was observed in control slices stained with only secondary antibody against anti-Drs B2 or anti-CD147, in presence of DAPI as visual exposition reference (panel 1A–1C). An additional control without Drs B2 treatment showed an absence of significant signal in the red channel, with a positive detection of CD147 in presence of DAPI (panel 1D–F). After treatment with 2.5 µM of Drs B2 during 1 hour almost all cells were labeled with the antibody but showed distinctive signals at different locations on or inside these cells.

Figure 5 panels 2A, 3A, 4A and 5A represent particular locations and different states of Drs B2 aggregation with a global maximum projection of the RGB composite stacks and their deeper views at different z levels (sub panels B–D). Among these different aspects, some small extra- or trans-membrane particles were captured (panel 2B–D). In other cases, these particles appeared as highly condensed structures (aggregates) contiguous to the extracellular cell membrane as is shown in panel 3B–D. Also visualized were cells wherein this condensed structure was enlarged and congregated within the membrane whereby non-homogeneous membrane labeling of CD147 suggests local membrane folds or disruption (panel 4B–D). This is a limitation in the low signal area for the precise location of the aggregates regarding the membrane position. Additionally, in certain cases, as for example the one in panel 5, Drs B2 staining was observed inside the nucleus. The last panel (panel 6) shows an idea of the significant representation of the Drs B2 aggregation in cell islets whereby four of the six cells presented were labeled. The panels 6B and 6C show selected confocal plans of aggregates at the cell surface (6B) and during cell penetration (6C). These aspects are also visible in the global volume rendering view of the whole cell group (6D). Remarkable is that in most cases, the Drs B2 aggregates were located at the proximity of the cell nuclei. Panels 2B–D, 3B–D, 4B–D, 5B–D and 6B–C showed the confocal location of the aggregates regarding the cell membranes and the nuclei, in some adjacent focal plans. The distribution of the aggregates at the cell surface as seen in 3D volume rendering pictures of panels 2E, 3E, 4E, 5E and 6D also displayed the form of these aggregates. In all these cases a part of the aggregates is deeply anchored in the cell membrane or is intra-cellular (panel 5E). It is noteworthy to mention that when PC3 cells were treated with a lower dose of Drs B2 (1 µM) or/and for different durations ranging from 1 to 30 minutes, similar cellular localizations of Drs B2 were observed. After 1 to 15 minutes of incubation with Drs B2, the small aggregates were predominant, and the frequency of events was higher when a dose of 2.5 µM was applied. With longer incubation times, the number of big aggregates increased and was higher for 2.5 µM Drs B2 (data not shown).

## Discussion

In this study we showed that Drs B2 displays a selective inhibition on the proliferation of all tested tumor adherent and non-adherent cell lines, with GI50 values in the low micromolar range. Also the proliferation of the non tumor but immortalized cells LB-EBV, HTK and PNT1A was inhibited. Interestingly, at a maximum tested concentration of 15 µM, Drs B2 did not affect the proliferation of the human primary cells FD and G1947. Drs B2 was also ineffective on primary cultures of mouse embryonic cells EMC and on NIH3T3 cell line (data not shown). These observations indicate that differences due to the long-life characteristics between tumor cells and normal non tumor cells could serve as a target of Drs B2. Noteworthy are the observed low GI50 values on all tested human tumor cells in contrast with those of other AMPs that have been reported to be cytotoxic for human tumor cells. For example the reported GI50s for magainins or gaegurins were in the 10 to 100 µM ranges. Similarly, a recent report has described Drs L1, a member of the Drs family isolated from Lemur leaf frog *Hylomantis lemur*, with a selective cytolytic activity against the human hepatoma cell line HepG2 (GI50 = 45 µM) [39].

Besides its inhibiting effect on cell proliferation *in vitro*, Drs B2 also inhibited PC3 and MDA-MB231 growth in an anchorage-independent manner and the resulting colony formation in soft agar in a dose dependant manner. However, tumorigenesis is a multistep process wherein also angiogenesis plays an important role in growth, progression and metastasis of all solid tumors. Therefore, the agents that inhibit angiogenesis could be effective in controlling primary growth and development of tumors as well as secondary metastatic tumors. In addition to the proliferation of primary endothelial cells ABAE and HUVEC, Drs B2 also significantly inhibited the differentiation of these endothelial cells on respectively collagen and Matrigel™ *in vitro*. However, no effect on both underlying endothelial monolayers was observed. These results would support the thinking that the effect of the peptide is dependent on the cell density. However, when testing the GI100 dose of Drs B2 on PC3 at full confluence, the effect of the peptide was still more than 50% (data not shown) suggesting that the cell density could not explain solely the effect of Drs B2.

In this study the inhibitory effect on tumor growth *in vitro* was confirmed by analyzing the potential therapeutic effect of Drs B2 in mice harboring experimental PC3 tumors. Drs B2 administrated at 2.5 mg/kg inhibited tumor growth with 50% compared to tumors treated with PBS. Additionally, in two mice out of eight the tumor was completely disappeared. Furthermore, the associated tumor angiogenesis was also inhibited by the Drs B2 treatment, confirming its angiostatic activity observed *in vitro*. Mice treated with Drs B2 behave normally and have a significant higher body weight compared to the PBS treated group (data not shown). To further evaluate its antitumor activity and any possible systemic toxicity, systemic routes of administration of Drs B2 need to be performed.

The differences in sensitivity of Drs B2 on tumor, non tumor and primary cells most likely reflect the differences in cell membrane composition through which this peptide acts. As described for the diverse activity of AMPs on microbial cells, it is preferable that these peptides do not pose the same activity on healthy eukaryotic cells. Fortunately, eukaryotic membrane composition significantly differs from those of prokaryotes that in addition have several characteristics similar to those of eukaryotic tumor cells. The negatively charged components of the cancer cell membranes, the greater membrane fluidity and cell-surface area were supposed to play an important role in attracting the cationic AMPs to the plasma membrane of the tumor cells like for the lytic activity of cationic AMPs on bacteria [12]. Therefore, it is very likely that the basic mechanism, by which AMPs act on microbes, is comparable to their activity on tumor cells.

Among the hundreds of AMPs isolated and studied so far, only a few were also investigated for their mode of action on cancer cells [40]. Most of those studies included biophysical techniques conducted mainly with model phospholipids membranes [17], [41] instead of directly on eukaryotic living cells. However, studies of AMPs on several types of cancer cells propose a wide variety of possible killing mechanisms *in vitro*. Some of these peptides destroy neoplastic cells by a similar cell membrane disrupting mechanism as is shown for bacteria whereas others cause destruction of mitochondrial membranes or/and induce cell death by caspase activation [12]. Even the use of one type of AMP on different cell types can lead to different proposed mechanisms. For example the well-studied bovine AMP LfcinB causes several cancer cells to die by apoptosis through a mitochondria and caspase-dependent pathway [42], [43], while other studies suggested that LfcinB kills cells by necrosis [44], [45].

The detailed mechanism by which Drs disrupt bacterial membranes, and, subsequently, inhibit their growth leading to cell death has not yet been provided. Data using simple artificial membranes and/or molecular dynamic simulations [46] led to the proposal that Drs bind to and permeate microbial cells through a carpet-like mechanism of action and that the N-terminal region of these peptides is mainly responsible for their membrane effects [47]–[50]. Recently, a more precise two step mechanism of Drs B2 mediated microbe membrane disruption was proposed [51] whereby the balance between charge, conformation, hydrophobicity, amphipathicity and polar angle due to flexibility was suggested to be very important for the antimicrobial activity of this peptide.

In the presented study the possible mechanism of cell death of PC3 prostate adenocarcinoma after Drs B2 exposure *in vitro* was evaluated by the data obtained from lethal concentration curves, assays testing cytoxicity, cell viability, mitochondrial membrane potential, expression of apoptotic markers and confocal microscopy analysis. Mainly, after exposure to cytotoxic drugs multicellular organisms employ cell death through the key mechanism apoptosis or through a less frequent manner by necrosis [52]–[56]. Since after Drs B2 treatment no PS exposure on the outer cell surface, no DNA fragmentation (data not shown), no changes in the mitochondrial transmembrane potential, and no caspase-3 activation were observed, it seems obvious that cells do not die by apoptosis. Contrary, the increased LDH release, the increased PI positive population and the confocal pictures showing binding to the plasma membrane, aggregation and entry of Drs B2 in the cells seems more convincing for the involvement of a necrotic-like pathway that may be triggered by the rupture of the plasmatic membrane. The exact molecular mechanism(s) by which Drs B2 kills PC3 cells is still not fully understood. It could be plausible that when comparing the activity of Drs B2 observed in this study, the necrotic-like symptoms on eukaryotic tumor cells resulted from an equal mechanism as is described on microbes. To clarify this point, further investigations will be needed. Interestingly, some studies with anticancer molecules, for example with Taxol®, mentioned that different ways of cell death could be induced depending on the used concentrations [57]–[59]. Additionally, small concentrations (8 µg/ml) of both α- and β-defensins enhance the proliferation of untransformed human epithelial cells and fibroblasts via the activation of MAPKs, whereas higher concentrations (50 µg/ml) of α-defensins are cytotoxic to these cell types [60]. Also in the presented study, a slight but significant increase in proliferation was observed in several cell types (HUVEC, MDA-MB231 and Raji) treated with low concentrations (<GI50) of Drs B2, but cell proliferation was decreased when increasing the peptide concentration (>GI50). Thus, also while studying Drs B2 different experimental conditions as substance concentration and time of exposure have to be evaluated before excluding cell death by apoptotic activation.

In conclusion, this study demonstrated that the natural derived AMP Drs B2 could be an interesting molecule for the treatment of cancer. Its antitumor and angiostatic activities, especially its selective targeting of tumor cells with micromolar concentrations propose Drs B2 as a potential candidate for the development of a new efficient targeting therapy against cancer. Further investigations are needed to clarify the selective killing activity and mechanism on tumor cells.

## Materials and Methods

### Materials

Dulbecco's modified Eagle's medium (DMEM) GlutaMAX containing 1 mg/L D-glucose and sodium pyruvate, DMEM GlutaMAX containing 4.5 mg/L D-glucose and sodium pyruvate, RPMI-1640 GlutaMAX, F12 containing L-glutamine, MCDB-131, fetal bovine serum (FBS), phosphate buffered saline (PBS), and Gentamycin (50 mg/mL) were from Gibco and provided by Invitrogen Life Technologies Corporation (Cergy Pontoise, France). Endothelial Basal Medium (EMB) and EMG-2 SingleQuots were from Clonetics provided by Lonza (Belgium). Bovine serum albumin (BSA) and agar were from Sigma-Aldrich (St Quentin en Fallavier, France), and trypsin (0.05% trypsin and 0.02% EDTA in Dulbecco's PBS (D-PBS)) was from PAA Laboratories (Les Mureaux, France). The BCA Protein Assay Kit was obtained from Pierce (Brebières, France), crystal violet was obtained from Gurr-Searle Diagnostic (High Wycombe; Bucks, England). Collagen type I was isolated in our laboratory, as described by Laaroubi et al. [61]. Recombinant human basic fibroblast growth factor-2 (FGF-2) was purified in the laboratory by sequential heparin-Sepharose and Mono-S chromatography from bacteria [62], [63]. Matrigel™ Basement Membrane Matrix was obtained from BD Biosciences (Bedford, UK). Drs B2 was synthesized as described previously [28]. Mouse monoclonal anti-CD34 was from Hycult Biotech (The Netherlands), secondary antibody, rabbit anti-rat IgG was obtained from Southern Biotech (Clinisciences; France). Mouse anti-human Ki67 antibody was from Tebu-bio (France) and biotinylated goat anti-mouse IgG was provided by Vector Laboratories (France). Anti-Drs B2 polyclonal rabbit serum was prepared by Marie-Claire Nevers and Christophe Créminon (Service de Pharmacologie et d'Immunologie, Gif-sur-Yvette Cedex, France). Secondary antibody, donkey anti-rabbit Fluo 546 was obtained from Interchim (Montlucon, France). Purified mouse anti-human EMMPRIN/CD147 was from BD Pharmingen (Bedford, UK) and goat anti-mouse FITC-AffiniPure was from Jackson ImmunoResearch Laboratories Inc. (West Grove, USA).

### Cell culture

PC3, DU145 (obtained from ATCC, catalogue number CRL-1435, HTB-81 respectively), PNT1A (obtained from Pr. F. Vacherot, Créteil, France, and described previously [64]) were cultured in RPMI supplemented with 5% (v/v) FBS. LNCap cells (obtained from ATCC, catalogue number CRL-1740) were cultured in RPMI supplemented with 10% (v/v) FBS. MDA-MB231 cells (obtained from ATCC, catalog number HTB-26) were cultured in DMEM 4.5 g/L supplemented with 10% (v/v) FBS. Raji (obtained from ATCC, catalog number CCL-86) and B-EBV-transformed cell line (LB-EBV, obtained from Dr. A. Achour, Paris, France, and described previously [65]) were cultured in RPMI supplemented respectively with 10% and 20% (v/v) decomplemented FBS. HTK, FD cells (both obtained from Dr. J. Jester, California, USA [66]) and adult bovine aortic endothelial (ABAE) cells were cultured in DMEM 1 g/L supplemented with 10% (v/v) FBS; ABAE cells were isolated and cultured as previously described [67]. Human umbilical vein endothelial cells (HUVEC; obtained from Lonza, Switzerland, catalog number C2519A) were cultured in EMB supplemented with EMG-2 kit according manufactories protocol. G1947 stromal cells were gift of Pr. F. Vacherot, Créteil, France. Cells were obtained from the non-neoplastic prostate tissue of patients undergoing cystoprostatectomy. Tissues were minced and digested in RPMI 1640 medium containing penicillin, streptomycin and fungizone supplemented with 10% FBS and 300 U/mL collagenase. Cells collected from the supernatant were plated and maintained in growth medium to establish primary cultures. After 2 weeks of growth, the stromal cells were separated from contaminating epithelial cells by differential rounds of trypsination. Informed consent forms were signed by patients prior to surgery and experiments were approved by the ethical committee of the Henri Mondor Hospital (Créteil, France). For experiments, cells were cultured in MCDB supplemented with 10% FBS and 1× ITS. Cells were cultured at 37°C in a controlled humidified environment with 7% CO_2_.

### *In vitro* proliferation assay

The cells were seeded at a density of 10^4^ cells/well in 24-well plates (1.91 cm^2^) in 0.5 mL complete medium and incubated at 37°C in a controlled humidified 7% CO_2_ environment. On the first, third, and fifth days after plating, the cells were treated with Drs B2 at different concentrations. Twenty four hours after the last treatment, adherent cells were washed with PBS1x, fixed with absolute ethanol, and cell counting was carried out with crystal violet staining (Gurr-Searle Diagnostic; High Wycombe; Bucks, England), as previously described [28], [68].

### Soft agar assay

PC3 or MDA-MB231 cells (10^4^ cells/well) were added to 1 mL of 0.35% top agar in complete medium containing Drs B2 and seeded onto a 0.8% agar bottom in 12-well plates (3.8 cm^2^). After polymerization, the agar/cell layer was covered with 0.5 mL of complete medium. Directly after seeding the cells and every forty eight hours during 10 days cells were treated with different concentrations of peptide. The number of colonies was determined as previously described [28].

### *In vitro* angiogenesis assay on collagen

Differentiation of the ABAE cell line induced by FGF-2 was tested on three-dimensional collagen type I gels prepared according to the Montesano procedure, with minor modifications [61]. In brief, 2×10^5^ ABAE cells per well of a 12-well plate (3.8 cm^2^) were seeded onto the collagen layer in complete medium supplemented with 5% FBS and FGF-2 (20 ng/mL). Immediately and 48 hours after plating, the cells were treated with 5 µM of Drs B2. Forty-eight hours after the last treatment, tubular network structures were observed and quantified, using an Axiovert 10 photozoom inverted microscope connected to a digital camera (Axiocam MRm Zeiss, Germany). Quantification of capillaries was determined by analysing five randomly chosen fields per well in triplicate. The results are expressed as the number of pseudo capillaries per field.

### *In vitro* angiogenesis assay on Matrigel

Differentiation of the HUVEC induced by FGF-2 was tested on Matrigel™ Basement Membrane Matrix (BD Biosciences, Bedford, UK). Briefly, 4×10^4^ HUVEC cells per well of a 24-well plate (1.91 cm^2^) were seeded onto a Matrigel™ layer (1/3 diluted) in complete medium supplemented with 2% FBS and FGF-2 (20 ng/mL). Immediately after plating, the cells were treated with different concentrations of Drs B2. Twenty four hours after cell plating, tubular network structures were observed and quantified, using an Axiovert 10 photozoom inverted microscope connected to a digital camera (Axiocam MRm Zeiss, Germany). Quantification of capillaries was determined by analyzing two randomly chosen fields per well in triplicate. The results are expressed as the number of pseudo capillaries per field.

### Tumor xenograft in nude mice

All *in vivo* experiments were approved by the appropriate French committee in charge of animal experimentation (DDPP) and conducted in compliance with European Community. Four weeks old female athymic nude mice (Janvier; Le Genest Saint Isle, France) were injected subcutaneously (s.c.) in the right flank with 2×10^6^ PC3 cells/100 µL RPMI1640. Three mice were not injected and were used as a wild type control group. After tumor development, mice were selected by tumor size and separated in three groups, so that each group has mice with ‘equal’ tumor sizes. Mice in the negative control group were treated six times per week peri-tumoral (p.t). with 100 µL of PBS. Mice in the experimental group were treated six times per week p.t. with 100 µL of Drs B2 at mentioned concentration. Twice per week the size of the tumors was measured along two major axes with a caliper. Tumor volume (mm^3^) was calculated with the formula: V = 4/3π*R_1_^2^ * R_2_ whereby radius 1 (R_1_) < radius 2 (R_2_). At the end of the experiment before sacrificing, mice body weights were observed and blood was collected by cardiac puncture. Tumors were obtained, covered with Tissue-Tek OCT (Sakura Finetek, France) and frozen directly into nitrogen liquid where after stored at −80°C.

### Proliferation and angiogenesis analyses of tumor xenografts by immunohistochemistry

PC3 xenografts were removed by surgery and immediately snap frozen in liquid nitrogen cooled isopentane. Frozen tumor sections of 6 µm thickness were prepared, fixed during 10 minutes in acetone at −20°C, rehydrated in PBS followed by blocking of endogenous peroxidase activity with H_2_O_2_ 1%. Tumor sections were washed twice with PBS and saturated in horse serum (Invitrogen Life Technologies Corporation, Cergy Pontoise, France) 5% (v/v) during 1 hour. Mouse anti-human Ki67 antibody (Tebu-bio, Le Perray en Yvelines, France) diluted in PBS/HS 1% (v/v) was added during 1 hour at 37°C in a humidity chamber. After two washes with PBS-Tween 20 0.2% (v/v), sections were incubated during 1 hour at room temperature with biotinylated goat anti-mouse IgG (Vector Laboratories; France) in PBS/HS 1% (v/v), followed by three washes and incubation with an avidin-biotinylated-alkaline phosphatase complex (Vectastain® Elite® ABC Kit, Vector Laboratories; France). Alkaline phosphatase activity was revealed using the DAB Substrate Kit for Peroxidase (Vector Laboratories, France). Finally, sections were counterstained with haematoxylin, and cover slipped with mounting medium.

For CD34 labeling, sections were prepared, fixed and blocked as mentioned above for Ki67 staining. Tumor sections were washed twice with Super Sensitive Wash Buffer (Biogenex; Fremont, CA, USA) and saturated in Power Block Universal Blocking Reagent (TRB; Biogenex; Fremont, CA, USA) during 10 minutes at room temperature. To visualize endothelial cells within the tumors, sections were incubated with mouse monoclonal anti-CD34 antibody (Hycult Biotech, The Netherlands) during 1 hour at 37°C in a humidity chamber. After two washes with TRB, sections were incubated in rabbit anti-rat IgG (Southern Biotech, Clinisciences; France) in saturation buffer, followed by two washes with TRB. Sections were incubated during 20 minutes in Poly-HRP anti-rabbit IgG (PowerVision poly-HRP IHC Detection Systems, Leica Biosystems; France), washed twice and incubated with 3,3′-Diaminobenzidine (DAB; Vector Laboratories; France) during 10 minutes. After three washing steps with distilled water, sections were counterstained with haematoxylin (Merck Millipore, France), and cover slipped with mounting medium (Thermo Shandon Inc, Pittsburgh, PA, USA).

Images were acquired using an Aristoplan Leitz microscope coupled to a CoolSNAP color CCD camera (Photometrics, Tucson, AZ, USA). For each stained section, six microscopic fields containing exclusively viable tumor cells were randomly selected for analysis. Mean values ± SEM were then computed for untreated and treated tumors. Image analysis was performed using an original program developed for the ImageJ software [69]. Prior to the analysis, image lightning was corrected using an acquisition of the light field of the optical path. A pre-treatment was first performed to improve color segmentation; no specific peroxidase staining signal was reduced using a color space transformation [70] and the background was removed using a band pass filter [71]. Color segmentation was then performed through an adaptive pixel color sorting, finding an optimal contrast between the red and blue channels using a dose-response model [72]. The images resulting from this step were then segmented using the default auto threshold method implemented into the ImageJ software [73]. The residual artifactual objects were removed by a final color sorting based on their red and blue channel histogram analysis [74].

### Protein determination

The quantification of total protein was performed using the microplate procedure according to the BCA Protein Assay Kit instructions (Pierce; Brebières, France). BSA was used as the standard [75].

### Lactate Dehydrogenase LDH Release Assay

Cells were grown in a 96 wells plate (1.500 cells/well/100 µL; culture medium with 5% FBS) and treated one time with 1 and 7.5 µM of Drs B2. Cellular membrane integrity was evaluated by measuring lactate dehydrogenase (LDH) activity released into the culture media after different time points of Drs B2 exposure. The CytoTox96 non-radioactive cytotoxicity assay (Promega; Charbonnières-les-Bains, France) was performed according manufactory instructions and quantified by measuring the absorbance at 490 nm. Cells were ruptured by the freeze-thaw method whereby the obtained total cell lysate was set as 100% LDH activity (total LDH). The experimental LDH release for each condition was calculated by the amount of LDH released in the medium over the total LDH concentration measured in the total cell lysate.

### Cell viability with FITC-Annexin-V/PI double staining

Cells were grown in a 6 wells plate (6×10^4^ cells/well/4 mL) and treated one time with Drs B2. Twenty four hours after exposure, cell viability was evaluated by flow cytometric analysis with FITC-Annexin-V/Propidium iodide (PI) double staining. Medium and trypsinized cells were collected and washed with PBS. After centrifugation cells were resuspended in PBS to obtain a cell density of 0.5×10^6^ cells per mL. One mL of this cell extract was centrifuged, resuspended in 200 µL PBS, transferred to a microtiterplate with round bottom and centrifuged again. Resulted cell pellet was resuspended in 200 µL of Binding Buffer 1× (Miltenyi Biotec, Bergisch Gladbach; Germany) containing 5 µL of FITC-Annexin-V (Miltenyi Biotec) and incubated during 10 minutes in dark at room temperature. The cells were washed with PBS where after incubated with 200 µL of Binding Buffer 1× containing PI (final concentration 1 µg/mL) (Miltenyi Biotec) during 5 minutes in dark at room temperature. Flow cytometric analysis was performed with MACSQuant Analyzer (Miltenyi Biotec).

### Mitochondrial membrane potential detection

Cells were grown in a 6 wells plate (2×10^5^ cells/well/3 mL) during 24 hours and treated one time with Drs B2. One and twenty four hours after exposure, changes in mitochondrial membrane potential were evaluated with flow cytometric analysis by using the lipophilic and cationic JC-1 dye (5,5′,6,6′-tetrachloro-1,1′,3,3′-tetraethylbenzimidazolylcarbocyanine iodide; Invitrogen Molecular Probes; Cergy Pontoise, France). Medium and trypsinized cells were collected, centrifuged where after the cells were washed with cold PBS. The pellet was resuspended in as much as needed pre warmed medium to obtain a cell concentration of 0.5×10^6^ cells/mL. The cells were stained with 2.5 µg/mL JC-1 dye dissolved in complete medium and incubated during 15 minutes in the dark at 37°C. Cells were washed twice with PBS, resuspended in 0.3 mL PBS where after analyzed with flowcytometer MACSQuant Analyzer (Miltenyi Biotec; Bergisch Gladbach, Germany). Cells treated with CCCP (50 µM) (Carbonyl cyanide m-chlorophenylhydrazone; Sigma-Aldrich; St Quentin en Fallavier, France) during 24 hours were used as positive control.

### Caspase activity assay

Cells were grown in a 6 wells plate (2×10^5^ cells/well/3 mL) during 24 hours and treated one time with Drs B2. Twenty four and forty eight hours after exposure, the cells were washed with PBS and plates were placed at −80°C during 24 hours. After, cells were defrozen during 10 minutes on ice. Lysis buffer was added, incubated during 5 minutes on ice and cells were scraped from the dishes and lysate was centrifuged at 13,000 g during 10 minutes. Protein content in cell lysate was determined. Isolates of different treated cell isolates were used to determine caspase activity. Caspase activity was assayed in a total volume of 200 µL per reaction. Briefly, 30 µg of protein was added to the reaction buffer (containing HEPES 30 mM, pH 7.4; EDTA 0.3 mM, pH 7.5; NaCl 100 mM; Triton X-100 0.15% and DTT 10 mM). The reaction was started by adding 10 µL of 2 mM of Ac-DEVD-AFC fluorogenic caspase-3 substrate (N-acetyl-Asp-Glu-Val-Asp-7-amino-4-trifluorometyl-coumarin (fluorogenic substrate in DMSO; Tebu-Bio; Le Perray en Yvelines, France). The plate was covered and mixed gently on a Titramax-100 plate shaker. The samples were incubated at 37°C in the dark during 2 hours and the reactions were stopped by cooling down the reactions on ice. The fluorescence was measured with a fluorescent micro plate reader (Fluoroskan II Labsystems iEMS Shadon; France) by an excitation and emission wavelength of 400 and 500 nm respectively.

### Immunofluorescent staining

PC3 cells were seeded on Matrigel™ pre-coated glass cover slips. Forty eight hours after cell plating, cells were treated with 2.5 µM of Drs B2 during 1 hour. Cells were washed with PBS, fixed with paraformaldehyde 4% during 5 minutes at room temperature. Cells were washed three times with PBS, where after unspecific sites were blocked with PBS containing 3% BSA, during 1 hour at room temperature. Cells were incubated with purified anti-Drs B2 polyclonal rabbit IgG (4 µg/mL in PBS/BSA 1%) over night at 4°C. After three times washing with PBS during 10 minutes for each wash, the secondary antibody, donkey anti-rabbit Fluo 546 (2.5 µg/mL in PBS/BSA 1%) was added and incubated during 1 hour at room temperature. For membrane visualization, cells were stained with purified mouse anti-human EMMPRIN/CD147 (1.6 µg/mL in PBS/BSA 1%) labeled with goat anti-mouse FITC-AffiniPure (7.5 µg/mL in PBS/BSA 1%). After five washes of 10 minutes with PBS, cells were incubated with DAPI-methanol (1 µg/mL) during 3 minutes, washed with methanol and air dried. Cover slips were mounted with Mowiol mounting solution.

Confocal fluorescence images were acquired using an IX81 inverted Olympus microscope equipped with a DSU spinning disk confocal system (Olympus; Rungis, France), coupled to an Orca R2 CCD camera (Hamamatsu Corporation; Japan). Observations were performed with the 60× objective (oil-immersion NA 1.25). Cells were analyzed by acquiring axial z stacks of confocal images (8 µm from the base to the top, with steps of 0.5 µm). Image processing was executed using the ImageJ software [69], and the Zoom in Images and Stack program [76]. Residual blurring were removed by spatial deconvolution: point spread functions (PSF) were calculated using the ImageJ's plugin PSF Generator [77], and the deconvolution, was performed using the Richardson-Lucy algorithm implemented into the DeconvolutionLab ImageJ's plugin [78]. These two last freeware programs are provided by the Biomedical Imaging Group of the EPFL (Ecole Polytechnique Fédérale de Lausanne; Switzerland). 3D rendering imaging was accomplished using the FreeSFP software (Scientific Volume Imaging B.V.; Hilversum, The Netherlands).

### Statistical analyses

Statistical analyses were performed using the GraphPad PrismTM version 4.00 software from GraphPad Software Inc. (San Diego, CA, USA). Results were expressed as their means ± standard deviation (SD) or standard error mean (SEM) of at least three determinations for each test from three independent experiments. Statistical analyses were carried out using the unpaired two-tailed t-test. The statistical significance of the differences is given as * p<0.05; ** p<0.01; *** p<0.001.
